# Two putative MmpL homologs contribute to antimicrobial resistance and nephropathy of enterohemorrhagic *E. coli* O157:H7

**DOI:** 10.1186/s13099-019-0296-7

**Published:** 2019-04-18

**Authors:** Salma H. Hussein, Reham Samir, Ramy K. Aziz, Mohamed A. Toama

**Affiliations:** Department of Microbiology and Immunology, Faculty of Pharmacy, Cairo University, Cairo, 11562 Egypt

**Keywords:** *Escherichia coli*, Efflux pumps, Secretome, Bioinformatics, RND, Fluoroquinolone sensitivity, Pathogenesis, Kidney damage

## Abstract

**Background:**

The serious human pathogen, *E. coli* serotype O157:H7, continues to gain antibiotic resistance, posing a public health threat. While this serotype’s genome has been sequenced, the role of 25% of its genes remains unknown, including genes conferring additional resistance. A prominent bacterial resistance mechanism is acquiring genes encoding efflux pumps, among which are the mycobacterial membrane proteins (Mmp), which contribute to virulence and membrane transport in mycobacteria. Here, we identified two potential *mmp* homologs (*z4861* and *yegN*) in *E. coli* O157:H7, and we aimed to investigate their distribution among *E. coli* strains and their potential functions.

**Methods and results:**

By screening different *E. coli* strains in vitro and in silico, we observed that *yegN* is more conserved than *z4861*. Using knockout mutants lacking either or both genes, we found that the mutants were more susceptible to fluoroquinolones than the parent strain and their secretomes included fewer virulence-related proteins. Moreover, histopathological examination of the kidneys of CD-1 mice infected by the wild-type or knockout strains indicated a greater impact of *z4861* on pathogenesis and kidney damage than *yegN*, since both mutants lacking *z4861* caused less severe kidney damage. The growth pattern of the wild-type was similar to that of mutant strains under aerobic and anaerobic conditions; yet, the mutant strains grew less when treated with subinhibitory dose of ciprofloxacin.

**Conclusion:**

The previously unannotated gene product, Z4861, and its more conserved homolog, YegN, contribute to the kidney damage and resistance of *E. coli* O157:H7.

**Electronic supplementary material:**

The online version of this article (10.1186/s13099-019-0296-7) contains supplementary material, which is available to authorized users.

## Background

*Escherichia coli* (*E. coli*) is a well-studied commensal member of the gut microbiota of humans and warm-blooded animals [1]. Most *E. coli* strains are harmless to humans, but a few pathogenic strains, classified into pathotypes, may cause severe health problems. For example, enteropathogenic *E. coli* (EPEC) is an important cause of life-threatening diarrhea in children; enteroinvasive *E. coli* (EIEC) is closely related to *Shigella*; and enterohemorrhagic *E. coli* (EHEC) causes severe foodborne diseases and frequent outbreaks [2–4]. The symptoms of these diseases usually include fever, vomiting, abdominal cramps and diarrhea that may progress to hemorrhagic colitis. EHEC infection may progress into life-threatening diseases, such as acute renal failure, hemolytic anemia and thrombocytopenia [5, 6].

Treatment of such life-threatening bacterial diseases is challenged by an alarmingly increasing resistance to commonly used antibiotics, such as tetracycline, erythromycin, and sulfamethoxazole. Moreover, a high level of resistance to ampicillin, amoxicillin–clavulanic acid, cefotaxime, imipenem, chloramphenicol, tetracycline, co-trimoxazole, nalidixic acid, ciprofloxacin, has been reported in different countries [7, 8] including Egypt [9, 10].

Antibiotic resistance in *E. coli*, like in other bacteria, is promoted by various mechanisms, such as inactivation or degradation of antibiotic molecules, target alteration or overexpression, and decreased intracellular concentration of the antibiotic by reduced uptake or active efflux. Antibiotic efflux is one of the most common resistance mechanisms in bacteria, and is promoted by efflux pumps, among which are the resistance, nodulation, and cell division (RND) transporters. A correlation between the overexpression of a prototype of RND efflux pumps and induction of spontaneous mutations within crucial genes that confer permanent antibiotic resistance has been described [11]. The RND protein family is well defined in several organisms, most notably in Gram-negative bacteria, e.g., *E. coli* and *Pseudomonas aeruginosa* [12]. RND pumps are large proteins that may reach up to 1100 amino acids. The inner membrane pump is formed of 12 transmembrane domains (TMD), and two extra-cytoplasmic loops emerging from the first and second TMD as well as the seventh and the eighth TMD. The extra-cytoplasmic loops were proposed to determine the substrate specificity for the pump. The driving energy for RND mediated efflux is obtained from proton motive force [13, 14].

RND pumps have multiple physiological roles, among which is the export of virulence factors, e.g., enterobactins and hemolysin [15, 16]. Moreover, they aid in the survival and colonization of *E. coli* inside the host through resisting the acidic pH of the gut and providing protection against the bactericidal effect of bile salts acids [17, 18]. RND proteins have been classified into several subfamilies that are haemolysin secretion protein D HlyD, lactococcin A secretion protein LcnD, RTX-I toxin determinant D, calmodulin-sensitive adenylate cyclase-haemolysin CyaD, colicin V secretion protein CvaA, Protease secretion protein PrtE, Alkaline protease secretion protein AprE, and several multidrug resistance proteins [19]. One recently discovered subfamily is the mycobacterial membrane protein Large (MmpL) family, first characterized in *Mycobacterium tuberculosis*. In *M. tuberculosis*, 13 genes encode RND proteins designated MmpL [20], with a major role in lipid transport, viability and virulence of the microorganism [21–24].

In *E. coli* strain K-12, the inner membrane protein (MdtB), which belongs to the multidrug RND efflux pump MdtABC, was found to have the same domains as MmpL proteins. The MdtABC pump is involved in the efflux of drugs, e.g., enterobactins, and bile salts [16, 25]. Although *E. coli*, especially the K-12 strain, is one of the most extensively studied microbes, the functions of over one-fifth of its genes remain unknown. Precisely, 23.5% of K-12 genes encode “hypothetical proteins” (according to the continuously updated SEED database [26, 27]). In pathogenic *E. coli*, the number of unknown genes is slightly higher (e.g., 25% of O157:H7 genes encode hypothetical proteins [28]). Thus, we sought to explore potential *mmpL* homologs among these genes.

To this end, we identified, through gene homology, two putative *mmpL*-like genes (*z4861* and *yegN*) in *E. coli* O157:H7. Whereas *yegN* is near identical to the K–12 *mdtB* gene, *z4861* has not been assigned a function. We studied the distribution of these two genes among different published *E. coli* genomes and clinical isolates, and we investigated their potential role in resistance and pathogenesis. We found that Z4861 and YegN contribute to the resistance of *E. coli* O157:H7 to fluoroquinolones. Moreover, the designed knockout mutants lacking either or both genes caused milder pathological kidney damage than the WT in a mouse model of infection.

## Results

### Distribution of *mmpL*-like homologs among *Escherichia coli* strains

**Fig. 1:**
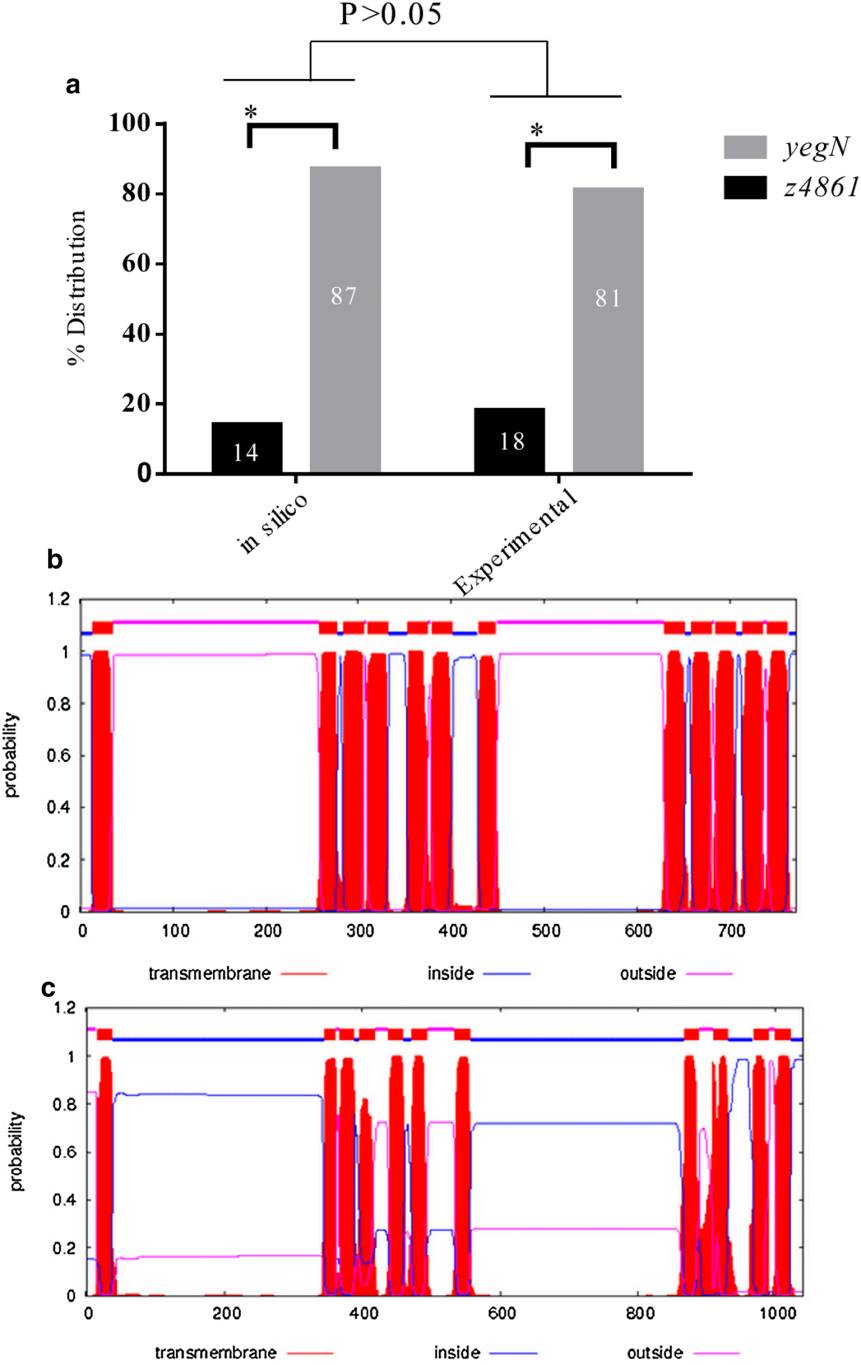
In silico and in vitro analysis of *z4861* and *yegN* and their products. **a** Distribution of *z4861* and *yegN* in published genome and clinical isolates. Relative percentile distribution of *z4861* and *yegN* in published *E. coli* genomes and in clinical isolates. No significant difference is observed between the distribution of both genes in silico and experimentally (*P* > 0.05), while the distribution of z*4861* significantly (*) differs from that of *yegN* either in silico or within *E. coli* clinical isolates (*P *< 0.05). **b**, **c** Prediction of membrane localization of Z4861 and YegN peptides, respectively. A typical presence of 11–12 transmembrane domains is observed, similar to the general structure of RND pumps. The analysis was performed on the TMHMM Server v. 2.0, with default settings

We first investigated the frequency of distribution of *z4861* and *yegN* genes in fully sequenced *E. coli* genomes and found that *yegN* is present more frequently than *z4861*. Both comparative genome analysis and in vitro screening of *E. coli* clinical isolates confirmed this unequal distribution (Fig. 1a), which was not associated with a particular pathotype.

Before experimentally investigating the potential cellular functions of these *mmpL* homologs, we analyzed their cellular localization and topology since RND proteins, by definition, are membrane proteins. Topology prediction of the products of these two genes revealed the presence of 11–12 transmembrane domains (Fig. 1b, c), a prediction that goes along with the general structure of RND pumps.

### The Δ*mmpL E. coli* O157:H7 mutants showed increased susceptibility to fluoroquinolone antibiotics

**Fig. 2:**
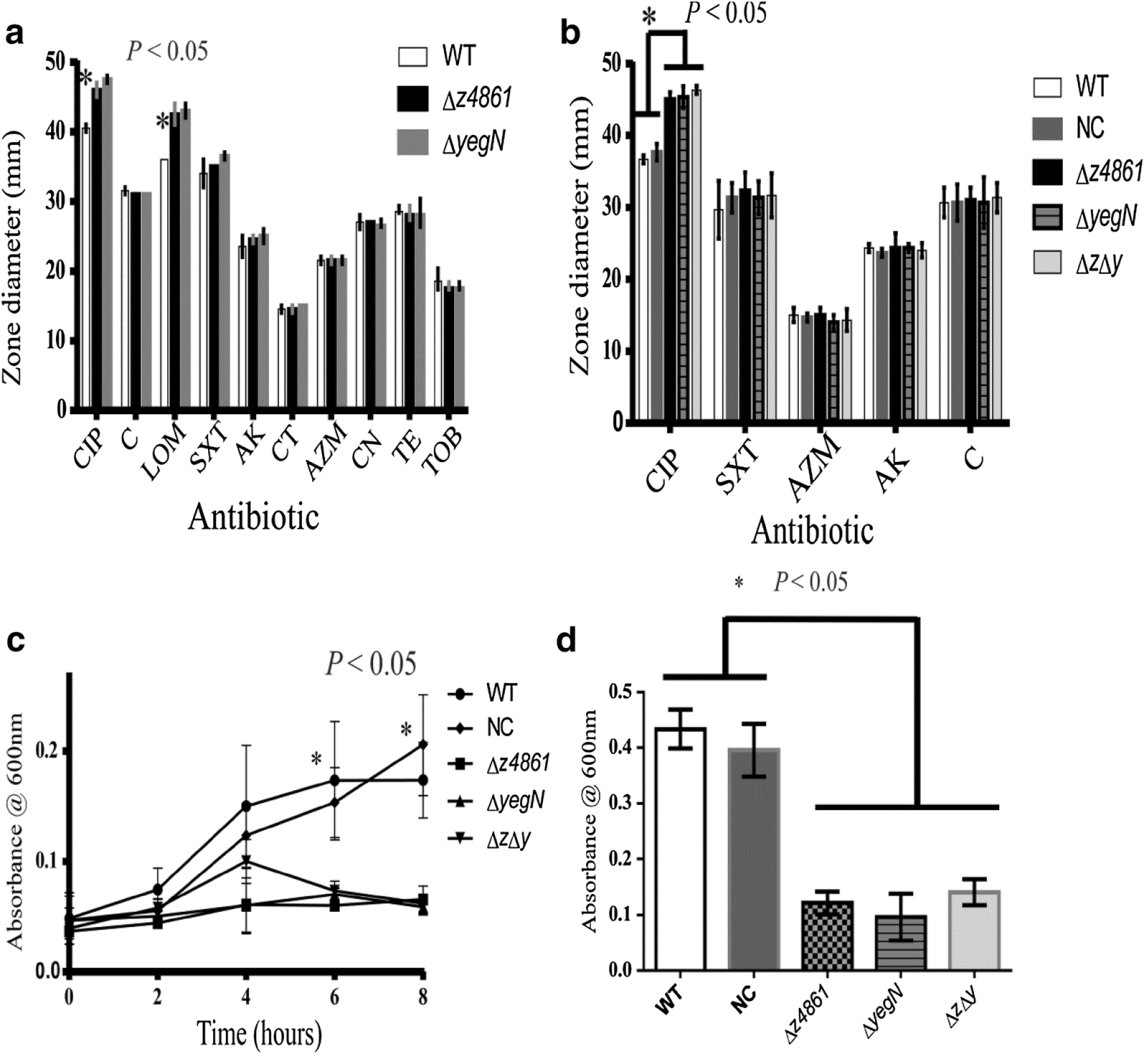
Effect of *yegN* and *z4861* deletion on antibiotic susceptibility. **a**, **b** Susceptibility of WT, Δ*z4861* and Δ*yegN* to different antibiotics belonging to different classes. A column chart of inhibition zone diameters (measured in mm) generated by different antibiotic discs applied to WT, Δ*z4861* and Δ*yegN* (**a**) or the same three isolates in comparison to the double mutant and NC (**b**). Significant differences in the mean zone diameters of the three strains were observed with the fluoroquinolones class represented by ciprofloxacin and lomefloxacin, while no significant differences were observed with other antibiotics (*P *>0.05). *CIP* ciprofloxacin, *C* chloramphenicol, *LOM* lomefloxacin, *SXT* sulphamethoxazole/trimethoprim, *AK* amikacin, *CT* colistin, *AZM* azithromycin, *CN* gentamicin, *TE* tetracycline, *TOB* tobramycin. The significance was determined by paired Student’s t-test. The data represent the means of three independent experiments and the error bars represent the standard deviation (SD). **c** Growth curves of the different mutants, WT, and NC strains in presence of sub-MIC concentration (0.0075 mg/l) of ciprofloxacin. No significant differences in the growth pattern of the five strains were observed till 4 h of incubation, but significant differences were observed between WT, NC and the three mutants starting from 6 h of incubation. No significant differences among the three mutants till 8 h of incubation were observed (*P *< 0.05). **d** Growth of the five strains, expressed at OD600 after 24 h incubation with sub-MIC concentration of ciprofloxacin. Significant differences were observed between the growth extent of WT, NC and the three mutants. No significant differences among the three mutants (*P *< 0.05). For **c**, **d**, analysis of variance (ANOVA) was used to estimate statistical significance. The data presented are the mean of three independent experiments, and the error bars represent the standard deviation (SD)

To investigate whether *z4861* and *yegN* might encode efflux pump proteins, we started by testing whether these gene products affect the susceptibility of *E. coli* to various antibiotics. To this end, we generated deletion mutants in *z4861, yegN,* or both and tested the resultant phenotype. Disc susceptibility testing against different antibiotics showed no difference between the WT and mutant strains, except with fluoroquinolones. All mutant strains were more susceptible to ciprofloxacin and levofloxacin than the wild type and NC strain (Fig. 2a, b).

### Growth of Δ*mmpL* mutants decreased upon exposure to sub-MIC of ciprofloxacin but not under anaerobic stress

**Fig. 3:**
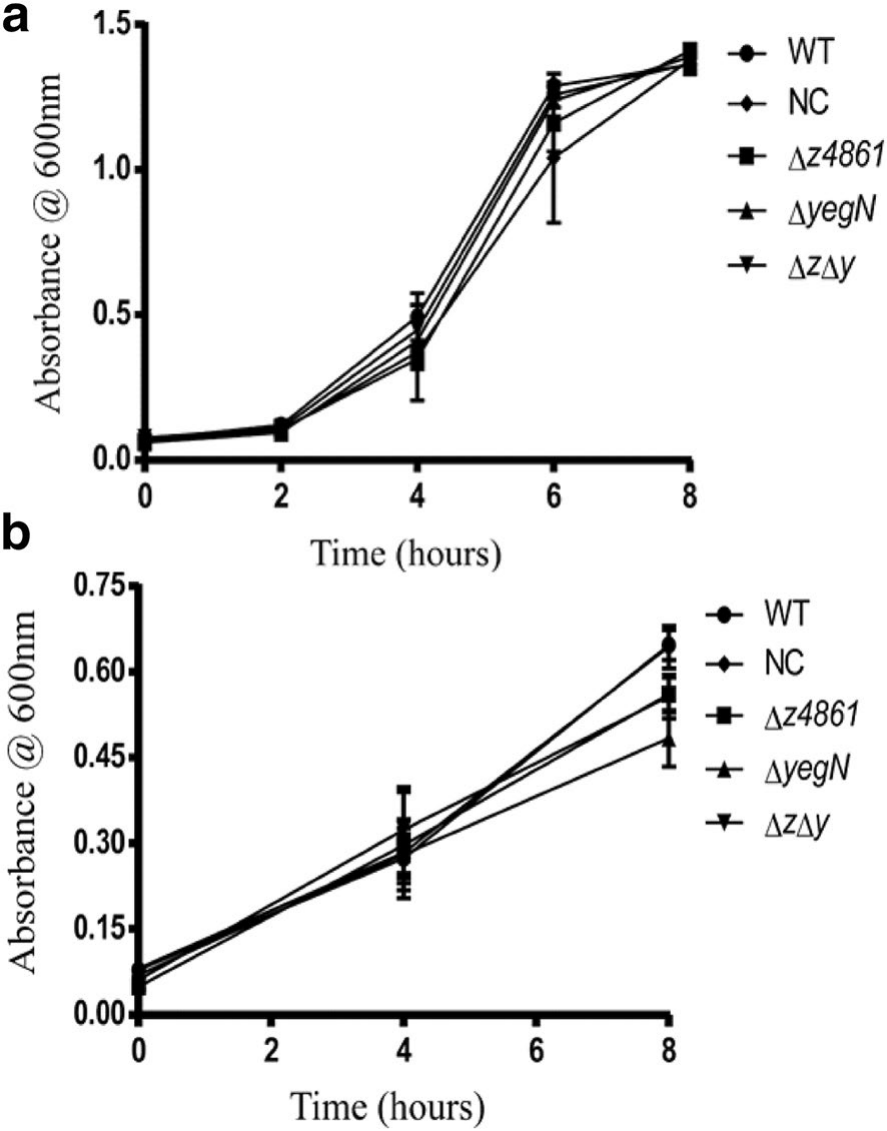
Growth curves of the WT, mutants and NC under aerobic and anaerobic conditions. No significant difference in the growth pattern of the five strains (measured as culture optical density) was observed under both aerobic (**a**) and anaerobic (**b**) conditions. The data presented are the mean of three independent experiments, and the error bars represent the standard deviation (SD). All values were tested by ANOVA for statistical significance

When allowed to grow in minimal salt media (MSM) supplemented with sub-MIC of ciprofloxacin, knockout mutants lacking either *z4861* or *yegN*, and the double mutant lacking both genes showed a significant decrease in growth after 6 h of inoculation (compared to the WT strain). The negative control (NC) strain showed similar behavior as the WT (Fig. 2c, d). On the other hand, the growth of the knockout mutants did not differ from that of the WT in absence of ciprofloxacin in both aerobic (Fig. 3a) and anaerobic (Fig. 3b) conditions.

### Transcription of *E. coli z4861* and *yegN* is upregulated upon treatment with sub-MIC of ciprofloxacin

Upon exposure to ciprofloxacin, the expression of *z4861* increased by a factor of 2.3–2.8, and the expression of *yegN* increased by a similar factor of 2.1–2.6 (Additional file 1: Figure S1).

### Secretome alterations in Δ*z4861* and Δ*yegN* mutants

**Fig. 4:**
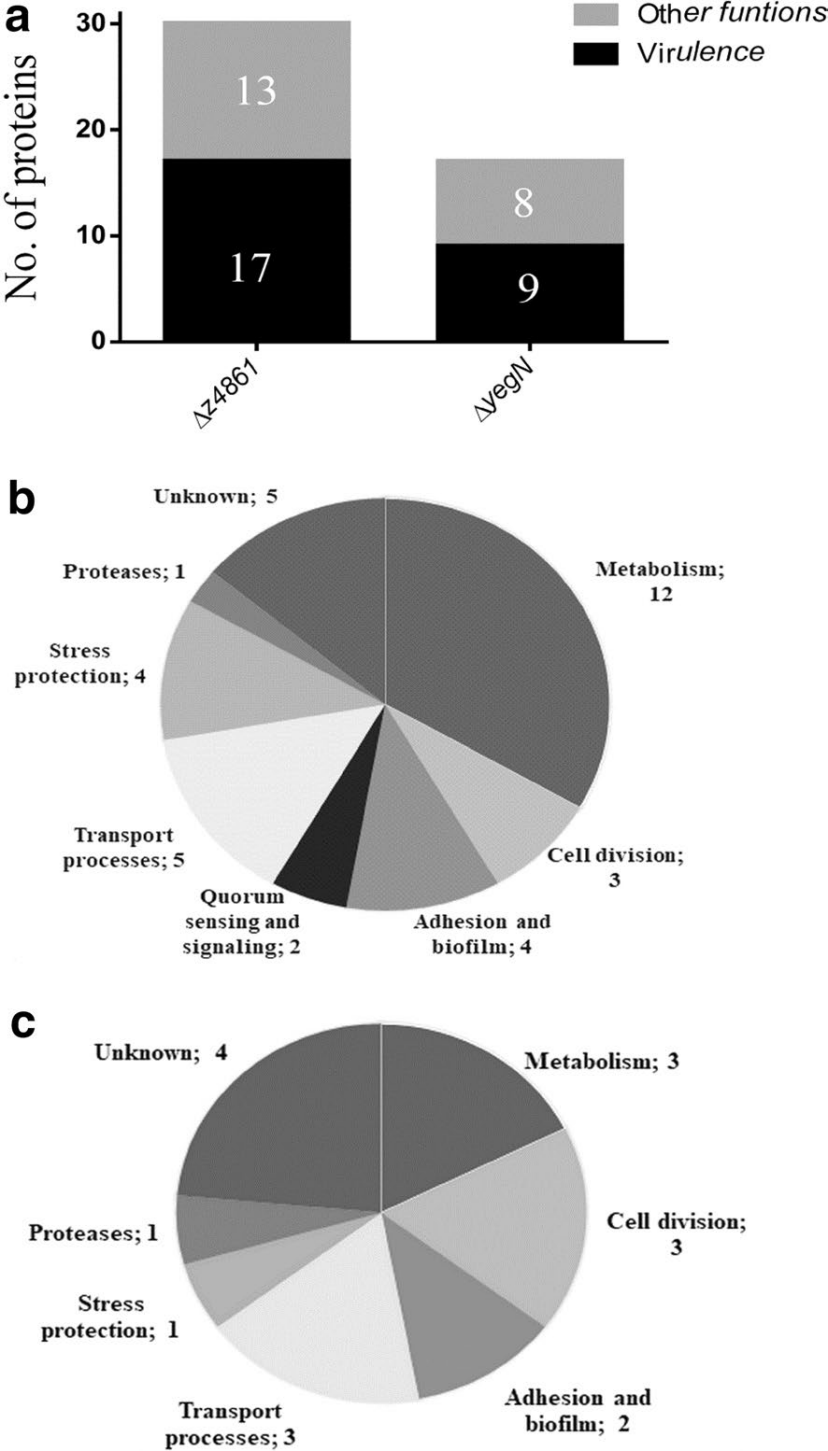
Secretome analysis. **a** Primary functional classification of differentially secreted proteins in Δ*z4861* and Δ*yegN* based on involvement in virulence or other cellular functions. The classification was done using VirulentPred [56]. **b**, **c** Functional categorization of affected proteins in Δ*z4861* and Δ*yegN,* respectively. This categorization was done using VICMpred [57]

Since MmpL proteins are involved in cell wall synthesis and export/efflux in *M. tuberculosis*, we set out to examine the effect of Δ*yegN* and *z4861* gene products on the secreted proteome (secretome) of *E. coli* O157:H7. When the secretome was analyzed by MALDI-TOF mass spectrometry, the number of absent or under-expressed secreted proteins (compared to WT) was 30 in case of Δ*z4861* and 17 in case of Δ*yegN*. The affected proteins were classified in two ways: First, they were classified into major functional classes, including virulence (Fig. 4a) and then they were categorized into subclasses.

This first classification showed that the deletion of *z4861* had a higher impact on the number of secreted proteins, with a higher proportion of virulence-related protein absent or present in less amounts. A second classification further categorized the affected proteins into functional subclasses, which showed that a number of proteins involved in prominent cellular and metabolic processes were affected in either or both mutants (Fig. 4b, c).

### Effect of knocking out *mmpL*-like genes on attachment to Caco-2 cells and biofilm formation

Assays concerning the adhesion of the WT, mutant, and the NC strains either on epithelial colon cells (Caco-2 cell line, Additional file 1: Figure S3) or on biofilm formation (Additional file 1: Figure S4) showed slight differences that were not statistically significant.

### The Δ*mmpL* mutants showed milder kidney damage in a mouse model of infection than the wild type strain

**Fig. 5:**
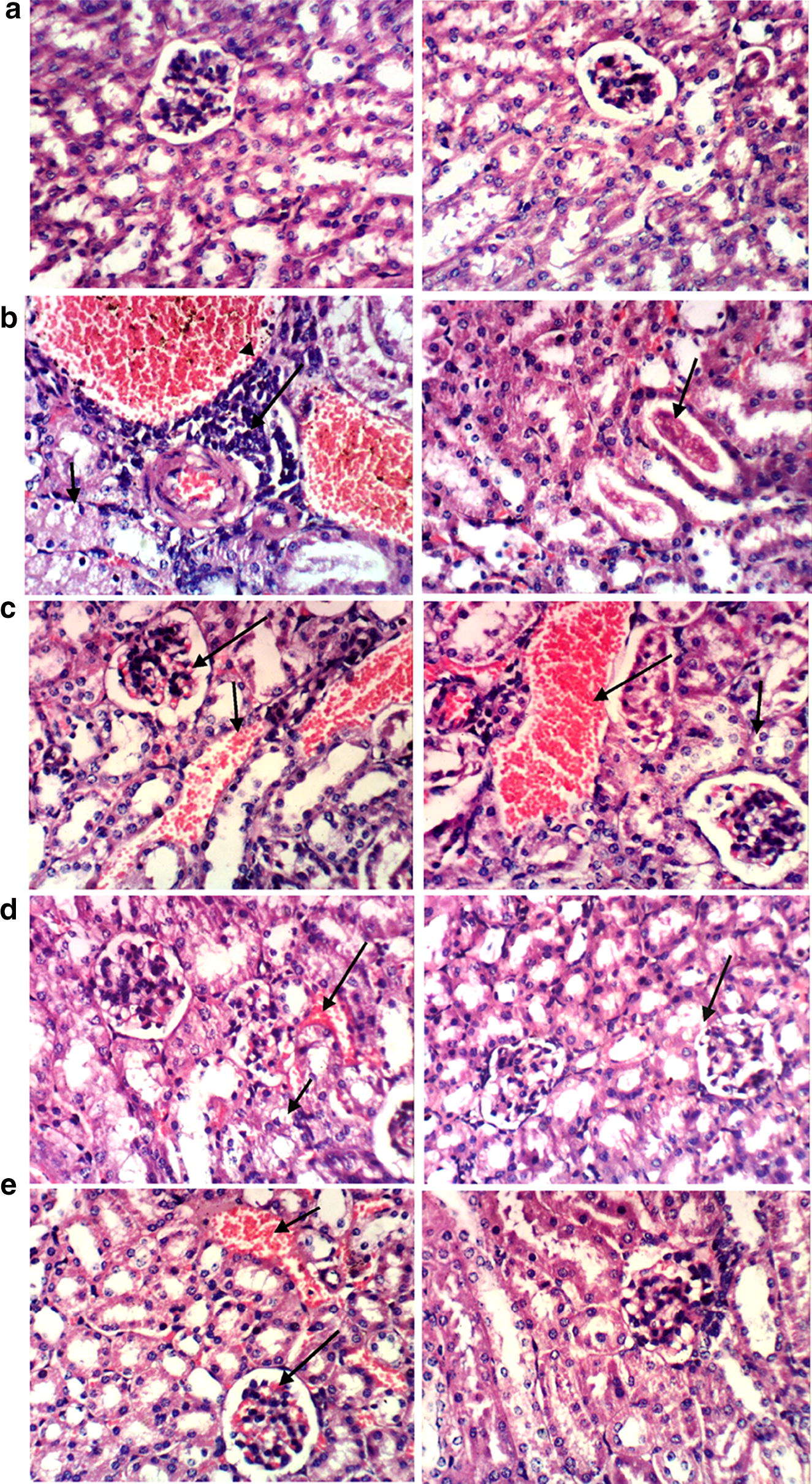
Histopathological analysis of H and E stained kidneys slides: Control and WT groups. **a** Control group: Both photos show the normal histological structure of renal parenchyma without any pathological features. **b** WT group: Vacuolation of renal tubular epithelium (short arrow), perivascular inflammatory cells infiltration (long arrow) and congestion of renal blood vessels (arrow head) (left), proteinaceous material in the lumen of renal tubules (arrow) (right). **c** Δ*yegN* group: Congestion of renal blood vessels (short arrow) and glomerular tuft (long arrow) (left), slight vacuolation of some renal tubular epithelium (short arrow) and congestion of renal blood vessels (long arrow) (right). **d** Δ*z4861* group: congestion of renal blood vessels (long arrow) (left), both photos show slight vacuolation of some renal tubular epithelium represented by the short arrow (left), or the arrow (right). **e** Δ*z*Δ*y* group: Congestion of renal blood vessels (small arrow) and glomerular tuft (large arrow) (left), while the right photo does not show any histopathological changes. Note that the entire experiment was repeated twice on two different sets of mice, and the presented results are collective of both trials

Histopathological examination of both kidneys demonstrated that each mutant strain caused kidney damage to a different extent (Fig. 5). The most dramatic kidney tissue damage was caused by the WT strain, followed—in descending order—by Δ*yegN*, Δ*z4861*, then Δ*z*Δ*y*, which caused the least tissue damage in comparison to the control group (Fig. 5 and Table 1).

**Table 1:** Evaluation of histopathological lesions caused by wild-type and mutant strains

Histopathological lesion	C	WT	*Δz4861*	Δ*yegN*	*Δz Δy*
Congestion of renal blood vessels	−	+++	+	++	+
Congestion of glomerular tuft	−	+++	+	+	+
Vacuolation of renal tubular epithelium	−	+++	+	+	±
Proteinaceous material in the lumen of renal tubules	−	++	−	−	−
Focal interstitial inflammatory cells infiltration	−	++	−	+	−

*C* control, *WT* wild-type strain; (−) no change, (+) mild change, (++) moderate change, (+++) severe change

## Discussion

*Escherichia coli* O157:H7 is one of the most notorious microbial pathogens with a long record of frequent outbreaks [2]. Since 1982, this strain has caused high morbidity and mortality among various age groups in USA and Europe through causing life-threatening conditions, such as HUS [29–31]. Its rapidly developing resistance against most antibiotics is a reason for concern and a motivation for serious search for novel drug targets.

Generally, bacteria can resist antibiotics by multiple intrinsic and extrinsic mechanisms, and *E. coli* O157:H7 is no exception. A distinct resistance mechanism in Gram-negative bacteria is pumping drug molecules outside the cell, thereby decreasing their intracellular concentration and rendering the usual therapeutic dose ineffective. Bacterial efflux pumps are classified into five major superfamilies, the most important of which in Gram-negative bacteria is the RND superfamily. Recently, some RND pumps were discovered in *M. tuberculosis* and were designated MmpL (mycobacterial membrane protein, large). These proteins are involved in many functions linked to cell wall synthesis, resistance and virulence inside the host [21].

Albeit initially discovered and studied in *M. tuberculosis* [32], proteins with the MMPL domain are widely distributed in Gram-negative, Gram-positive and acid-fast bacteria. Yet, MmpL homologs neither perform the exact functions nor share a high percentage of sequence identity [21–23, 25, 33] which makes the discovery of novel members of that family challenging.

The RND pumps described in Gram-negative organisms generally tend to be transcribed as operons, this operon arrangement was observed for *yegN*. On the other hand, the operon, in which *z4861* lies, is currently annotated to be involved in lipid metabolism and transport, a function that is consistent with the general properties of Mmpl-like gene products. Moreover, the genes encoding the Membrane Fusion Protein (MFP) and the inner protein may not be adjacent, and more than one RND pump can use the same OM channel [34, 35].

To investigate the possible role of *mmpL* homologs in *E. coli* O157:H7, we generated single and double deletions of these genes, and tested their: (i) aerobic and anaerobic growth, (ii) susceptibility to different classes of antibiotics, and (iii) role in fitness through potential involvement in protein transport and virulence.

(i) *Aerobic and anaerobic growth on the mutants*

First, no significant growth differences under aerobic conditions were observed among the mutant strains or between any of the mutants and the WT strain (Fig. 3a). This observation rules out that the mutagenesis process had any drastic effect on the bacterial cell viability, nor did it select for any growth-deficient auxotrophs.

Taking advantage of the wealth of publicly available genome-wide experimental data, we queried transcriptomic data available in the NCBI Gene Expression Omnibus (GEO [36]). GEO microarray studies of *E. coli* K-12 showed that *mdtB* (*yegN* homolog) was slightly overexpressed during aerobic-to-anaerobic shift. Thus, we investigated the growth pattern of WT and all constructed strains under anaerobic conditions but found no statistical differences in their growth patterns (Fig. 3b). The disagreement between the anaerobic growth results and the published microarray data could simply be due to strain-specific regulatory differences between O157:H7 and K-12. It is also possible that even if there were changes in *yegN* transcription under anaerobic conditions, this transcriptional change would not directly affect growth.

(ii) *Susceptibility to different classes of antibiotics*

The conserved domains in both proteins and their predicted localization across the cell membrane (Fig. 1b, c) suggested that *z4861* and *yegN* might encode RND pumps. To test this hypothesis, we analyzed the susceptibility of the mutants, as well as the WT and the NC strains, to antibiotics representing different classes, which are either routinely used for treatment of *E. coli* infections or that are known substrates for RND efflux pumps. The only observed difference in the susceptibility of the mutant strains was against fluoroquinolones (Fig. 2).

This difference in susceptibility was mirrored by a significant increase in the number of transcripts of *z4861* and *yegN* after the WT strain was grown in presence of sub-MIC of ciprofloxacin (Additional file 1: Figure S1). This finding is supported by a microarray study of the transcriptome of *E. coli* K-12 that confirmed an increase in expression of *mdtB* when subjected to norfloxacin [37].

It is to be noted that the observed changes in antibiotic resistance are different from what was reported about other members of the MmpL family. In other bacteria, members of this family conferred resistance to isoniazid and oxadiazoles [22, 23]. This incongruity is not fully surprising as protein family members often express different phenotypes, or at least different specificities, in different organisms. Additionally, even homologous proteins may be folded differently in a different organism. Finally, homology is often limited to one or a few domains but not to entire proteins. Thus, the differences in antibiotic susceptibility could be due to the different nature of *E. coli* cell wall from that of *M. tuberculosis* or *S. aureus*, or the possible different substrate specificity of these predicted efflux pumps, which may result in similar efflux activity but on different antibiotics or different substrates.

(iii) *Role of the MmpL homologs in fitness through potential involvement in protein transport and virulence*

Although the in vitro biofilm formation ability of the WT and mutant strains and their attachment to Caco-2 epithelial cells were not significantly different (Additional file 1: Figures S3 and S4), combining the secretome analysis data with previously published data about MmpL homologs suggested a possible effect on virulence. This discrepancy necessitated using a suitable animal model of infection to investigate whether these genes offer an overall fitness advantage in vivo.

In addition, RND pumps play an important role in the survival of *E. coli* inside the gut through resisting bile salt the acidic pH thus maintaining homeostasis [38]. So, mutations in RND pumps are directly related to the colonization ability of *E. coli* and its survival in the host.

The animal infection model used in this study suggests that *mmpL* gene products may be involved in virulence, and that this involvement was only revealed inside the host, since in vitro assays did not show significant phenotypic changes.

Whether the effect on virulence and kidney pathology is caused by a direct involvement of these proteins in the transport of virulence proteins or through an indirect role in the regulation of some of the major virulence factors (e.g., Shiga toxins) remains to be investigated. At this stage, we tried to rule out that the knockout mutants had different role on phage induction or on Shiga toxin expression, and we have not found any in vitro differences. However, we cannot rule out in vivo differences in Shiga toxin expression and that can be triggered by the interplay between bacterial and host factors. Future studies with advance in vivo imaging and expression analysis models would be invaluable.

Interestingly, *z4861* is located on one of the O-islands differentiating *E. coli* O157:H7 from *E. coli* K-12, and was found to be downregulated by the stationary phase Sigma factor (RpoS), together with several genes encoded on the same O-138 island [39]. This reported observation bolsters the hypothesis that this gene’s product may be involved in virulence, since many of the O-island-encoded genes are believed to contribute to pathogenesis, notably that genes on this island (O-138) seem to be co-expressed [39].

Of note, because of technical limitations, the deleted genes were not re-introduced in frame in the knockout strains (genetic complementation). Genetic complementation is usually conducted to rule out that any observed phenotypic differences could be due to other random, coincidental, or polar mutations. However, the fact that the phenotypic changes, such as fluoroquinolone resistance, were similar in three different strains (∆*yegN, Δz4861* and *ΔzΔy*) strengthens our hypothesis that both genes, not other factors, are behind the phenotypic shifts in the knockout strains. Since all mutations were precisely introduced in-frame by full replacement of the deleted open reading frames (from ATG to stop codon), polar effects are ruled out. If the observed fluoroquinolone resistance were due to an inadvertent genetic effect (e.g., a second unintended mutation or the selection of a natural mutant during the electroporation process), this phenotype would not have been consistent in all genetic constructs except the WT strain. Additionally, we introduced a negative control (NC) strain, which is a WT strain that underwent all the processes to which the mutants were subjected, such as competent cells preparation, electroporation (without any genetic material), and recovery. Designing and using this NC strain aimed to rule out any phenotypic changes that would have occurred during the physical preparation of the mutants.

Finally, the histopathological examination of the animal model’s kidneys consistently and reproducibly showed a relatively similar reduction in kidney tissue damage with Δz4861 and ΔzΔy, but not ∆yegN, suggesting that this phenotypic change is attributed to the deletion of *z4861*. This particular gene product may be involved in some virulence mechanisms. For example, Z4861 protein may play a role in the efflux of bile salts, which play an important role in enteropathogenic *E. coli* and EHEC by modulating the expression of specific virulence factors involved in the adhesion to epithelial cells [40]. Alternatively, the observed phenotype may simply be due to the failure to efflux certain signaling molecules that play an important role in virulence.

## Conclusion

In conclusion, this study aimed to investigate the role of two *E. coli* gene products, YegN and Z4861, in a representative of the clinically important but less studied O157:H7 EHEC pathotype. Different bioinformatics tools indicated a possible role of these gene products as efflux pumps of the MmpL family, their transmembrane localization, and a higher conservation of *yegN* than *z4861* in published *E. coli* strains. The latter finding was confirmed in clinical isolates. Upon deletion of each or both genes from a representative O157:H7 strain, the knockout strains were less resistant to fluoroquinolones, and *z4861* mutants were particularly less able to damage kidneys than the parent strain. On the other hand, no difference was observed in aerobic or anaerobic growth, biofilm formation ability, attachment to Caco-2 cell lines, or susceptibility to most other antibiotics.

This study is a first step towards a better understanding of the role of these two predicted proteins in particular, and MmpL-like proteins in general, in *E. coli.* Future studies will address the specific roles of each of these gene products, their substrate specificity and binding, as well as the specific mechanisms by which they alter bacterial susceptibility to fluoroquinolones and kidney damage.

## Methods

### Ethics statement

All animal procedures followed the guidelines of the Research Ethics Committee of the Faculty of Pharmacy, Cairo University. The research protocol was revised and approved by that committee.

### Bacterial strains and culture conditions (Additional file 1: Table S1)

*Escherichia coli* O157:H7 EDL933 was used in all the experiments as the wild-type (WT) strain, from which the knockout mutants and negative control (NC) strains were derived. The NC strain is a WT strain subjected to all physical conditions to which the mutants were exposed, namely all steps of competent cell preparation as well as electroporation.

All strains used in this study were grown in LB broth with shaking at 180 rpm, or on LB agar plates (Difco, USA) at 37 °C. When needed, LB was supplemented with kanamycin (50 mg/l) or ampicillin (100 mg/l). The WT and mutant strains were also grown in M9 glucose minimal salt media (MSM) which was prepared as described in Sambrook’s manual [41].

MacConkey and Eosin Methylene Blue agar plates were used for confirmation of identity and purity of *E. coli* laboratory strains and clinical isolates.

### Plasmids

Plasmids pKD46, pKD13 and pCP20 were used for targeted gene deletion by the Lambda Red system according to the protocol of Datsenko and Wanner [42] as modified by Baba and coworkers [43].

### Bioinformatics analyses

Amino acid sequences of MmpL and its homologs were retrieved from the NCBI database. Additional homologs were collected by BlastP database searches [44], and their sequences were compared by the alignment tool of CLC Main Workbench 5 (Qiagen, Valencia, CA, USA). Percentage identity and similarity of different aligned proteins were calculated by EMBOSS 6.3.1: matcher Waterman–Eggert local alignment [45]. The topology of Z4861 and YegN across the membrane was predicted according to Krogh et al. [46]. Secreted proteins were predicted by SignalP [47].

### Screening *E. coli* clinical isolates for the presence of *z4861* and *yegN*

Clinical isolates were screened for the presence of *z4861* and *yegN* by the polymerase chain reaction (PCR). Primers covering the conserved regions among different *E. coli* strains were used (Additional file 1: Table S2). Representative PCR products were confirmed by sequencing after agarose gel purification (Qiagen, Germany).

### Construction of the deletion mutants

The Lambda Red system was used for generating the knockout mutants [42, 43]. Briefly, a kanamycin resistance cassette, flanked by 57 nucleotides that are homologous to the 57 nucleotides immediately upstream or downstream the target gene plus the start and the stop codon, respectively, was used to replace the target gene. For the confirmation of the correct insertion of the kanamycin cassette and subsequent removal from the chromosome, PCR with specific primer pairs (Additional file 1: Table S2 and [48]) was used followed by Sanger sequencing of PCR products (Macrogen, Korea).

### Real time RT-PCR analyses of *z4861* and *yegN*

Bacterial cells were grown to midlog phase in M9 MSM, with or without supplementation with 0.0075 mg/l ciprofloxacin. RNA was isolated by the RNeasy kit (Qiagen, Germany). The concentration and purity of the extracted RNA were checked by a nanophotometer (P330 nanophotometer Implen, Germany).

Qiagen RT PCR kit was used for first cDNA, and a Quantitect SYBR Green PCR kit (Qiagen, Germany) was used for Real-time PCR. Equal aliquots of cDNA derived from RNA samples were used as templates in the amplification reactions. The housekeeping gene ihfB [49] was chosen as the control for the normalization of cDNA loading in each PCR. An aliquot of 1 µg of DNase-treated RNA was added in each reaction as a non-reverse transcribed control. Reactions were performed in Rotor-Gene Q (Qiagen, Malaysia), and the fold change in the levels of the transcripts was determined by the ∆∆Ct method [50].

### Mouse model of infection

We implemented a previously described oral infection model for *E. coli* O157:H7 [51]. Briefly, four groups of six to 8-weeks-old CD-1 male mice (Theodor Bilharz Research Institute, Egypt) were fed orally with an infection dose of 10^10^ CFU of each strain suspended in LB broth containing 20% sucrose, in addition to a control group given LB broth with no bacterial cells. All mice were euthanized 7 days post-infection.

Four mice from each group were dissected, and their kidneys were isolated and subjected to further histopathological examination. For histopathological examination, each isolated kidney was fixed in 10% neutral buffered formalin for 72 h. After fixation, the kidneys underwent gradual dehydration and were embedded in paraffin. The paraffin wax blocks were sectioned into 5 µm thick slices, and stained with hematoxylin and eosin (H & E) stain [52]. Each kidney was examined and compared to the kidneys of the control group. They were scored according to the extent of different pathological features.

### Proteomic analysis

Twenty-five milliliter of overnight LB cultures of each strain was normalized to the same OD_600_ absorbance. Supernatants of equal volumes of each culture were filtered through 0.22 μm cellulose acetate syringe filters. The bacterial supernatants were concentrated to a final volume of 500 μl in 9 K MWCO protein concentrators (Pierce, UK). Fractions were analyzed on 15% SDS-PAGE gels, stained with Coomassie blue, and then visually inspected (Additional file 1: Figure S2).

For the secretome analysis, the concentrated proteins were loaded on 10% polyacrylamide gels, stained, and the gel lanes were then excised and washed. Excised gel pieces underwent trypsin digestion [53]. Identification of proteins was performed in the Taplin Mass Spectrometry Facility, Harvard Medical School by matrix-assisted laser desorption/ionization time-of-flight (MALDI-TOF) mass spectrometry, as explained in [54].

### Attachment assay

Monolayer Caco-2 cells were incubated with 4 × 10^7^ CFU of WT or mutant bacteria in duplicate wells. After incubation at 37 °C for 2 h, each well was gently washed with PBS five times to remove any unattached bacterial cells. For cell detachment, 100 µl of 0.25% trypsin EDTA was added to each well and incubated for 20 min at 37 °C [55]. Following detachment, the samples were tenfold diluted, and 10 µl of each dilution was spotted on LB agar plates for viable cell count.

For estimation of cell attachment percentage, the total CFU recovered was divided by the initial inoculum, as follows: $\frac{\text{recovered bacterial count}}{\text{initial inoculum}} \times 100.$

### Antibiotic susceptibility tests

Antibiotic susceptibility tests were performed according to the British Society for Antimicrobial Chemotherapy (BSAC) disc diffusion method protocol. Susceptibility tests were performed in three biological replicates, and inhibition zones were measured in two different directions, then their mean was recorded in millimeter.

### Growth experiments

Growth experiments in M9 glucose MSM were performed in triplicates. All strains were grown overnight in LB broth, then pellets were harvested, washed, and suspended in M9 MSM to a final OD_600_ = 1. The adjusted bacterial suspensions were diluted 1:50 in M9 MSM (either plain or supplemented with ciprofloxacin at a sub-MIC of 0.0075 mg/l). Based on the preliminary twofold serial dilution experiment, this particular concentration was found to be the sub-MIC concentration which allowed the WT and the mutant strains to grow and in the same time allowed differences between them to be detected). The cultures were incubated with shaking at 37 °C and 180 rpm. The OD_600_ of the samples were measured at 0, 2, 4, 6, 7, 8, and 24 h.

### Statistical analyses

GraphPad Prism version 5.01 (GraphPad Software, La Jolla, CA, USA) was used for all statistical analyses. A *p* value threshold of 0.05 was implemented for statistical significance estimation.

## Additional file

**Additional file 1: Table S1.** Bacterial strains used in this study. **Table S2.** List of primers used in this study. **Figure S1.** Transcriptional changes of the *z4861* and *yegN* mutants grown in MSM with and without ciprofloxacin. **Figure S2.** SDS-PAGE analysis of WT, *Δz4861* and *ΔyegN* culture supernatants. **Figure S3.** Attachment assay on Caco-2 cell line. **Figure S4.** Biofilm assay of the five strains.
