# Supplementary material for: Two putative MmpL homologs contribute to antimicrobial resistance and nephropathy of enterohemorrhagic E. coli O157:H7
Source: Gut Pathog. 2019 Apr 18;11:15. doi: 10.1186/s13099-019-0296-7 (PMC6471949; doi:10.1186/s13099-019-0296-7)
Supplement: Supplementary file 1 — Additional file 1: Table S1. Bacterial strains used in this study. Table S2. List of primers used in this study. Figure S1. Transcriptional changes of the z4861 and yegN mutants grown in MSM with and without ciprofloxacin. Figure S2. SDS-PAGE analysis of WT, Δz4861 and ΔyegN culture supernatants. Figure S3. Attachment assay on Caco-2 cell line. Figure S4. Biofilm assay of the five strains. [file 13099_2019_296_MOESM1_ESM.pdf]

Two putative MmpL homologs contribute to antimicrobial resistance and nephropathy of enterohaemorrhagic *E. coli* O157:H7

Salma H. Hussein, Reham Samir, Ramy K. Aziz, Mohamed A. Toama

Department of Microbiology and Immunology, Faculty of Pharmacy, Cairo University, 11562 Cairo, Egypt

Additional File Contents:

- **Additional Tables:**

  Table S1: Bacterial strains used in this study

  Table S2: List of primers used in this study

- **Additional Figures:**

  Figure S1: Transcriptional changes of the *z4861* and *yegN* mutants grown in MSM with and without ciprofloxacin

  Figure S2: SDS-PAGE analysis of WT, *Δz4861* and ΔyegN culture supernatants.

  Figure S3: Attachment assay on Caco-2 cell line

  Figure S4: Biofilm assay of the five strains

Table S1: Bacterial strains used in this study

Strain	Genotype or description	Source or reference
Escherichia coli O157:H7 EDL933	Wild type (WT)	[1]
WT/pKD46	WT containing lambda phage plasmid pKD46; ampicillin resistant.	This study
Δz4861	Isogeneic mutant of WT lacking *z4861* gene; kanamycin resistant.	This study
ΔyegN	Isogenic mutant of WT lacking *yegN* gene; kanamycin resistant.	This study
ΔyegNΔkan	Isogenic mutant of WT lacking *yegN* gene and *kanamycin* insert.	This study
ΔyegNΔkan/pKD46	Isogenic mutant of WT lacking *yegN gene* and carrying lambda phage plasmid pKD46; ampicillin resistant	This study
ΔzΔy	*yegN* and *z4861* (double) mutant of WT lacking both *z4861* and *yegN*; kanamycin resistant	This study
E. coli clinical samples	Isolated from various hospitals and clinics	Department of Microbiology Culture Collection

Table S2: List of primers used in this study

Name	Site of binding	Sequence	Note	Source
K1'	Kanamycin cassette	5'-CCAGTCATAGCCGAATAGCCT-3'	Screening for the kanamycin cassette in the chromosome	[2]
K2	Kanamycin cassette	5'-CGGTGCCCTGAATGAACTGC-3'		[3]
Kt	Kanamycin cassette	5'-CGGCCACAGTCGATGAATCC-3'		
SH_001	*Z4861*	5'-CCTGCTGCCGCAATCACG-3'	Transcriptional analysis of *z4861*	This study
SH_002	*z486l*	5'-CGGCATCCATTGGCCCTTTA-3'		This study
SH_003	*yegN*	5'-CCCGTCGCGCCTGTTTATT-3'	Transcriptional analysis of *yegN*	This study
SH_004	*yegN*	5'-GCCAGACATCTGCCCGAACT-3'		This study
SH_005	Upstream *z4861*	5'-GCGATCGCACCGATATTGCTCTTACCCAACATCAACTGACGCCAGCGCAACTGACCGATGattccggggatccgtcgacc-3' *	Formation of *z4861* knockout insert	This study
SH_006	Downstream *z4861*	5'-AGCCGGTGAGGATAAGCGTAGCGGTCAGGGCGAACGCTCGCCAGAATGTGGATTTGATCAgtgtaggctggagctgcttc-3' *		This study
SH_007	Upstream *yegN*	5'-CACTCCGGAAGAGAAAGCCACCAGCCGCGAATACGCGAAAAAAGGAGCTCGCTCCTGATGattccggggatccgtcgacc-3' *	Formation of *yegN* knockout insert	This study
SH_008	Downstream *yegN*	5'-GACCGACAGTAAAATCGTCGCCACCGGGCGGTAAATGAAGAGGGCAAAAAACTTCACTTAgtgtaggctggagctgcttc-3' *		This study
SH_009	150 bp upstream *z4861*	5'-TCAACACCATCGATCTCGCC-3'	Confirmatory primers for *z4861* deletion	This study
SH_010	150 bp downstream *z4861*	5'-GATGCGCGTGCCAGGTTG-3'		This study
SH_011	150 bp upstream *yegN*	5'-CATTGATCGCCTGACCGAAG-3'	Confirmatory primers for	This study

Name	Site of binding	Sequence	Note	Source
SH_012	150 bp downstream *yegN*	5′-GCAGCATACGGAAACCCAGTA-3′	*yegN* deletion	This study
SH_013	*z4861* fw	5′-GATGGACGGTTGGCTGGTGA-3′	*z4861* samples screening	This study
SH_014	*z486l* Rev	5′-TGAGCAGCGCCAGTAGCAA-3′		This study
SH_015	*yegN* Fw	5′-TGCGGTGCAGGCAATTATGG-3′	*yegN* samples screening	This study
SH_016	*yegN* Rev	5′-GCCGACCATGCCGATACCTAA-3′		This study
ihfB Fw		5′-GATAGAAAGACTTGCCACCCA-3′	House Keeping gene	[4]
ihfB Rev		5′-CCAGTTCTACTTTATCGCCAG-3′		

* Nucleotides shown in lowercase represent the sequence homologous to the pKD46 plasmid, but not the target strain genome

Figure S1:

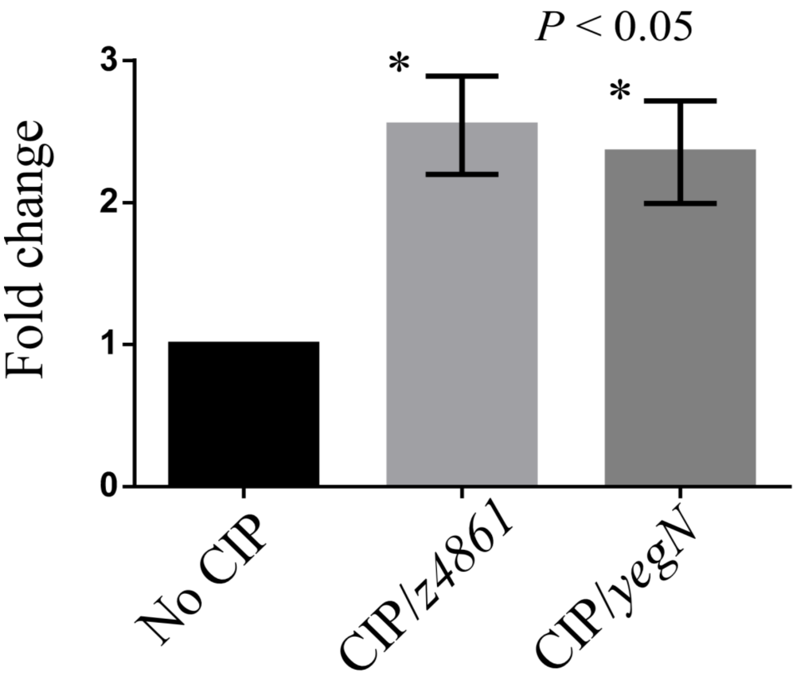

Figure S1: Transcriptional changes of the z4861 and yegN mutants grown in MSM with and without ciprofloxacin.

Fold change in the transcription levels of the *z4861* and *yegN* genes in both absence and presence of ciprofloxacin: A significant increase of 2.3-2.8 fold (in case of *z4861*) and 2.1-2.6 fold (in case of *yegN*) is observed when either mutant is grown in presence of ciprofloxacin. The data represent the mean of two independent experiments (each one was done in duplicates), and the error bars represent the standard deviation (SD).

Figure S2:

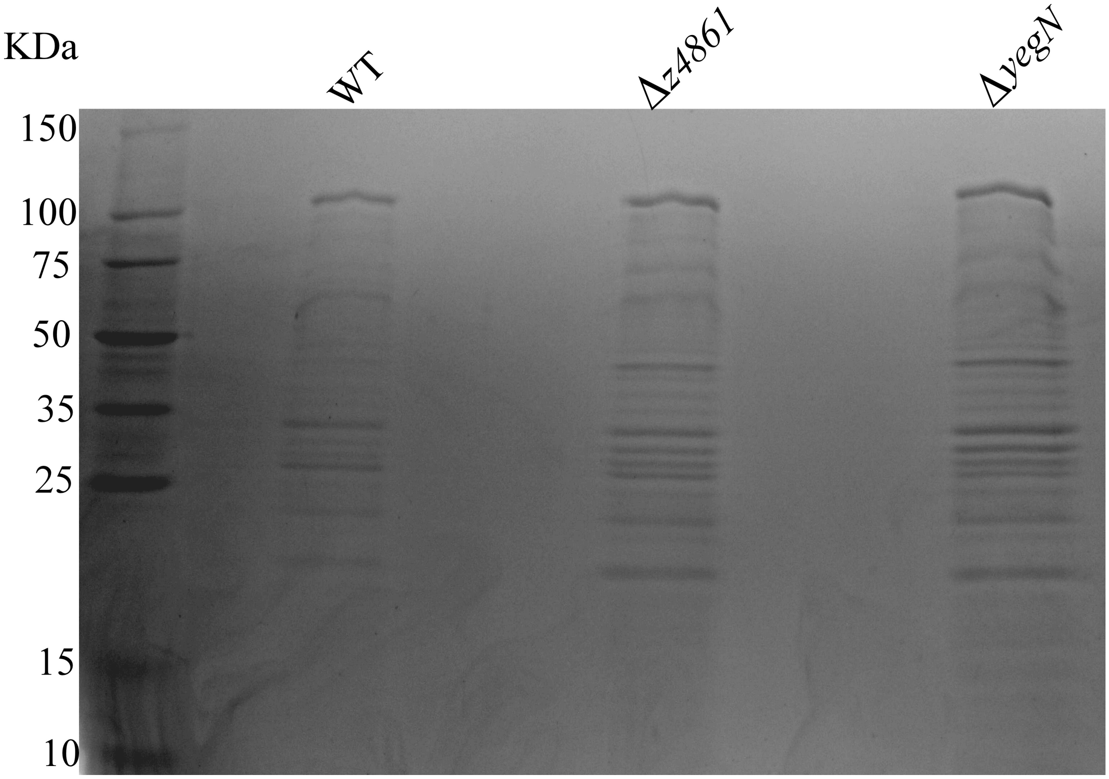

Figure S2: SDS-PAGE analysis of WT, Δ*z4861* and Δ*yegN* culture supernatants.

A 15% SDS-polyacrylamide gel showing the concentrated proteins for WT, Δ*z4861* and Δ*yegN*. No major differences could be visually detected between the analyzed secretomes.

<u>Figure S3</u>:

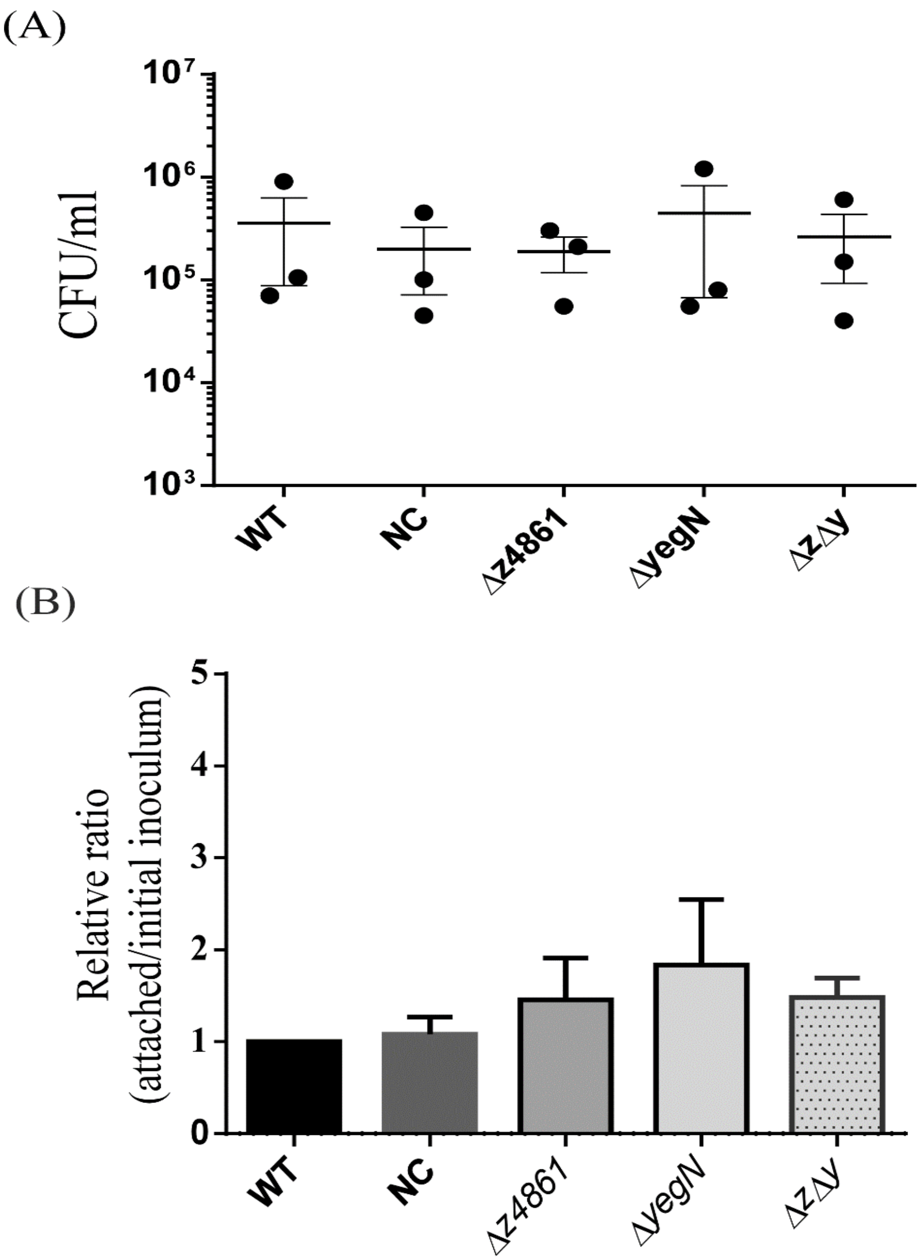

Figure S3: Attachment assay on Caco-2 cell line.

A. Graph of $\log_{10}$ CFU of recovered attached cells on Caco-2 cells for each strain. It shows no significant difference among the recovered cells for each strain ($P > 0.05$). **B.** Column chart comparing the ratio of recovered attached cells per initial inoculum of WT to that of each strain. The WT is given a default value of 1. No significant difference was found among the strains. The significance was determined by paired Student's t-test.

Figure S4:

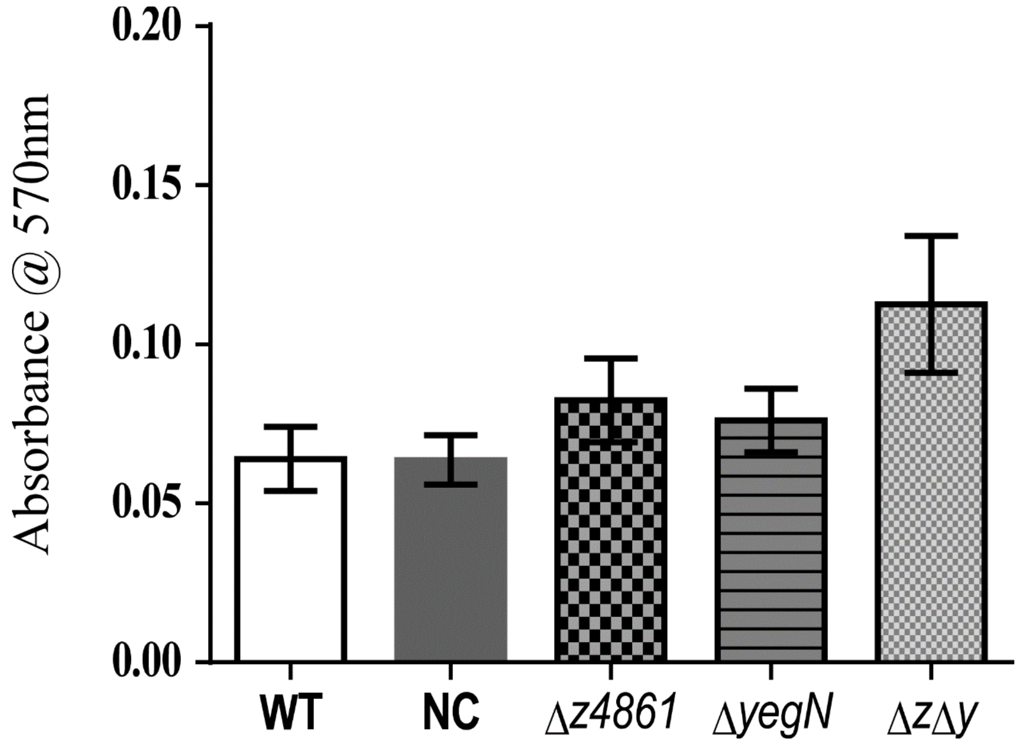

Figure S4: Biofilm assay of the five strains.

A column plot showing the absorbance of crystal violet stained biofilm of each strain. No statistical significance was found among the strains ($P > 0.05$). The significance was determined by pairwise multiple t-tests. The data presented is the mean of three independent experiments and the error bars represent the standard deviation (SD).

References for Additional Material:

1. Latif H, Li HJ, Charusanti P, Palsson BO, Aziz RK. A gapless, unambiguous genome sequence of the enterohemorrhagic *Escherichia coli* O157:H7 strain EDL933. Genome Announc. 2014;2. https://doi.org/:10.1128/genomeA.00821-14.
2. Aziz RK, Khaw VL, Monk JM, Brunk E, Lewis R, Loh SI, et al. Model-driven discovery of synergistic inhibitors against *E. coli* and *S. enterica* serovar Typhimurium targeting a novel synthetic lethal pair, *aldA* and *prpC*. Front Microbiol. 2015;6:958. https://doi.org/:10.3389/fmicb.2015.00958.
3. Datsenko KA, Wanner BL. One-step inactivation of chromosomal genes in Escherichia coli K-12 using PCR products. Proc Natl Acad Sci U S A. 2000;97:6640-5. https://doi.org/:10.1073/pnas.120163297.
4. Zhou K, Zhou L, Lim Q, Zou R, Stephanopoulos G, Too HP. Novel reference genes for quantifying transcriptional responses of Escherichia coli to protein overexpression by quantitative PCR. BMC Mol Biol. 2011;12:18. https://doi.org/:10.1186/1471-2199-12-18.
